# Supplementary figures and images for: Chromosome level assembly and comparative genome analysis confirm lager-brewing yeasts originated from a single hybridization
Source: BMC Genomics. 2019 Dec 2;20:916. doi: 10.1186/s12864-019-6263-3 (PMC6889557; doi:10.1186/s12864-019-6263-3)

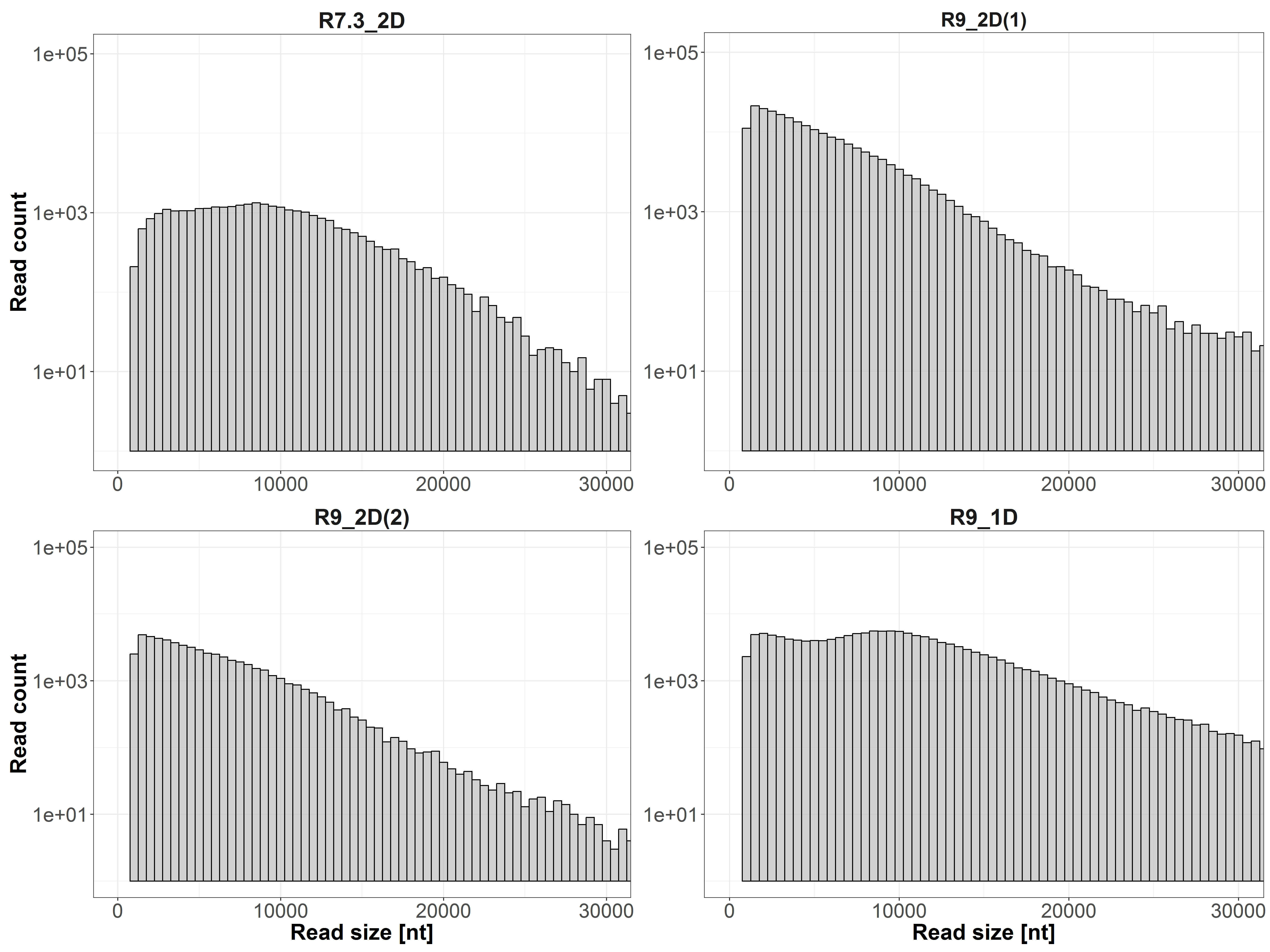

Supplement: Supplementary file 2 — Additional file 2: Figure S1. Read-length distribution obtained of the long-read libraries of CBS 1483 produced in this study. This plot shows the four different sequencing libraries obtained from whole genome sequencing of CBS 1483 using the MinION® platform. The Y-axes the frequency and the X-axes depict read-lengths (capped at 30 Kbp) using bins of 500 bp. The libraries were obtained with different sequencing chemistries due to the rapid development of Oxford Nanopore MinION sequencing. [file 12864_2019_6263_MOESM2_ESM.png]

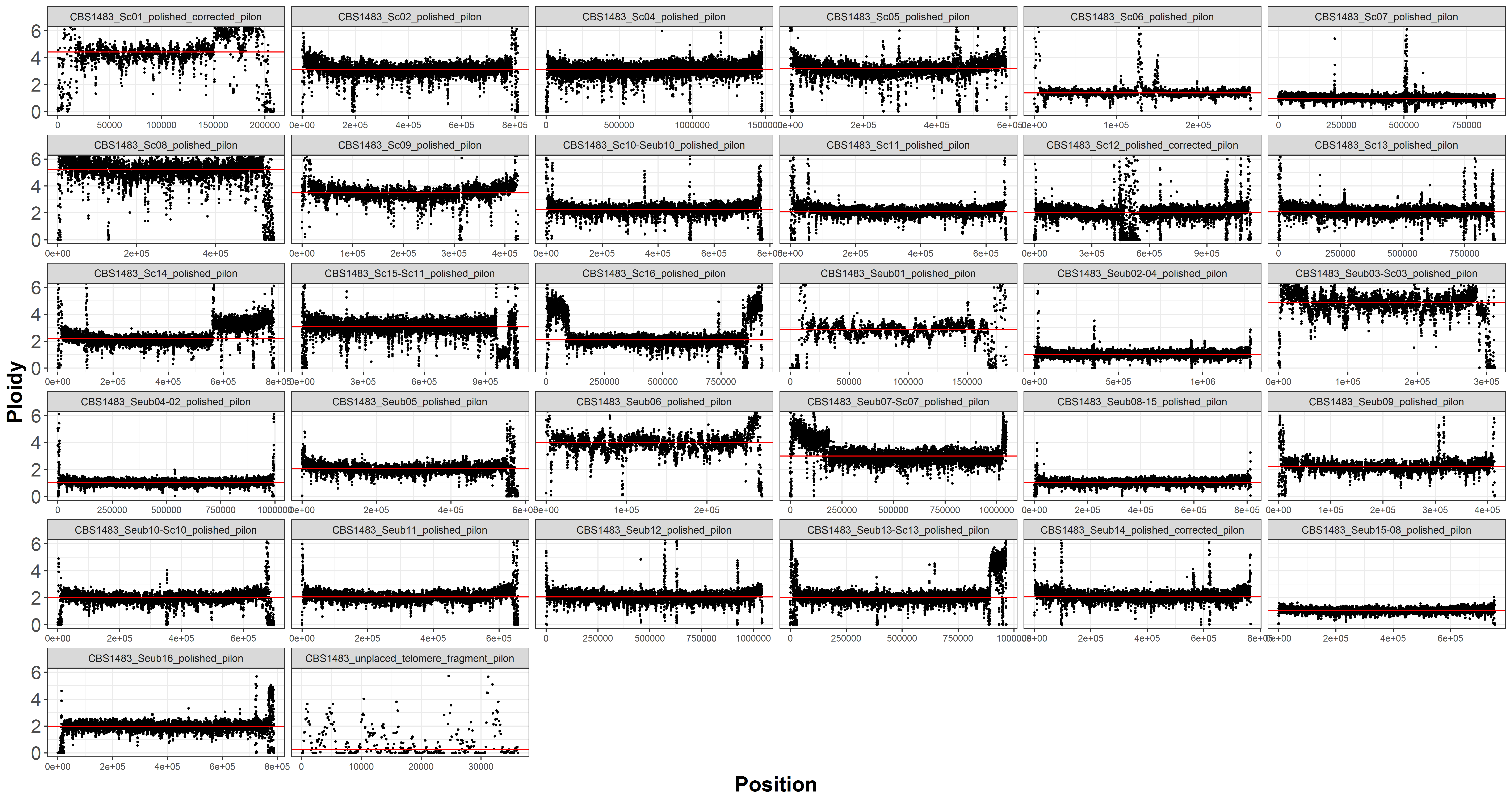

Supplement: Supplementary file 3 — Additional file 3: Figure S2. The ploidy estimates for each chromosome in the CBS 1483 assembly based on Illumina short read data from this study. The red horizontal lines correspond to the median coverage of each chromosome. [file 12864_2019_6263_MOESM3_ESM.png]

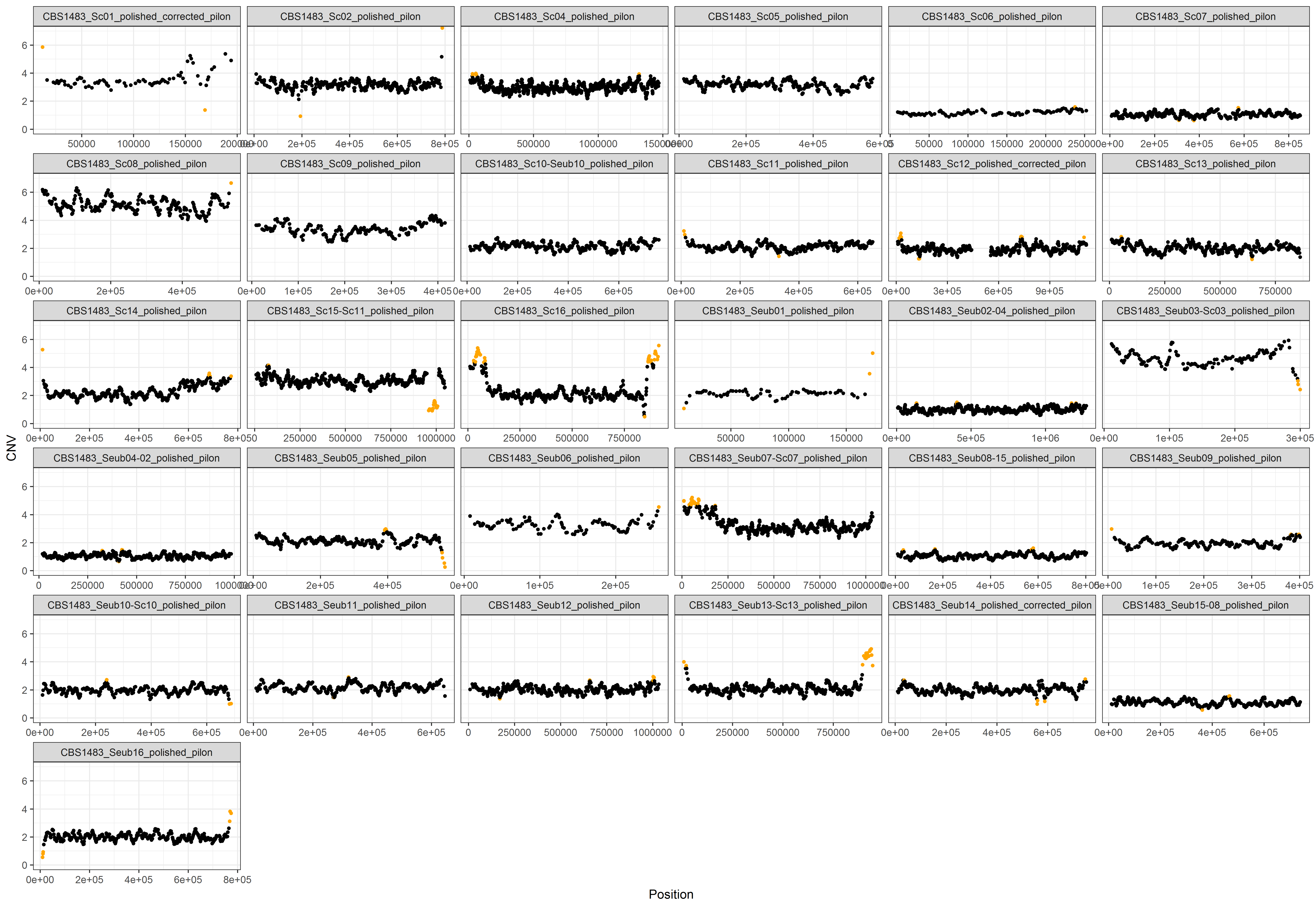

Supplement: Supplementary file 4 — Additional file 4: Figure S3. Copy number predictions for ORFs in the genome assembly of CBS 1483. Each dot represents an ORF whose x-value represents the location in the corresponding chromosome and the y-value the estimated copy number based on coverage analysis of long-read-only alignments. Dots in yellow indicate ORFs whose coverage significantly deviates from the surrounding genomic region, indicating copy number variation of the ORF within different copies of the same chromosome. [file 12864_2019_6263_MOESM4_ESM.png]

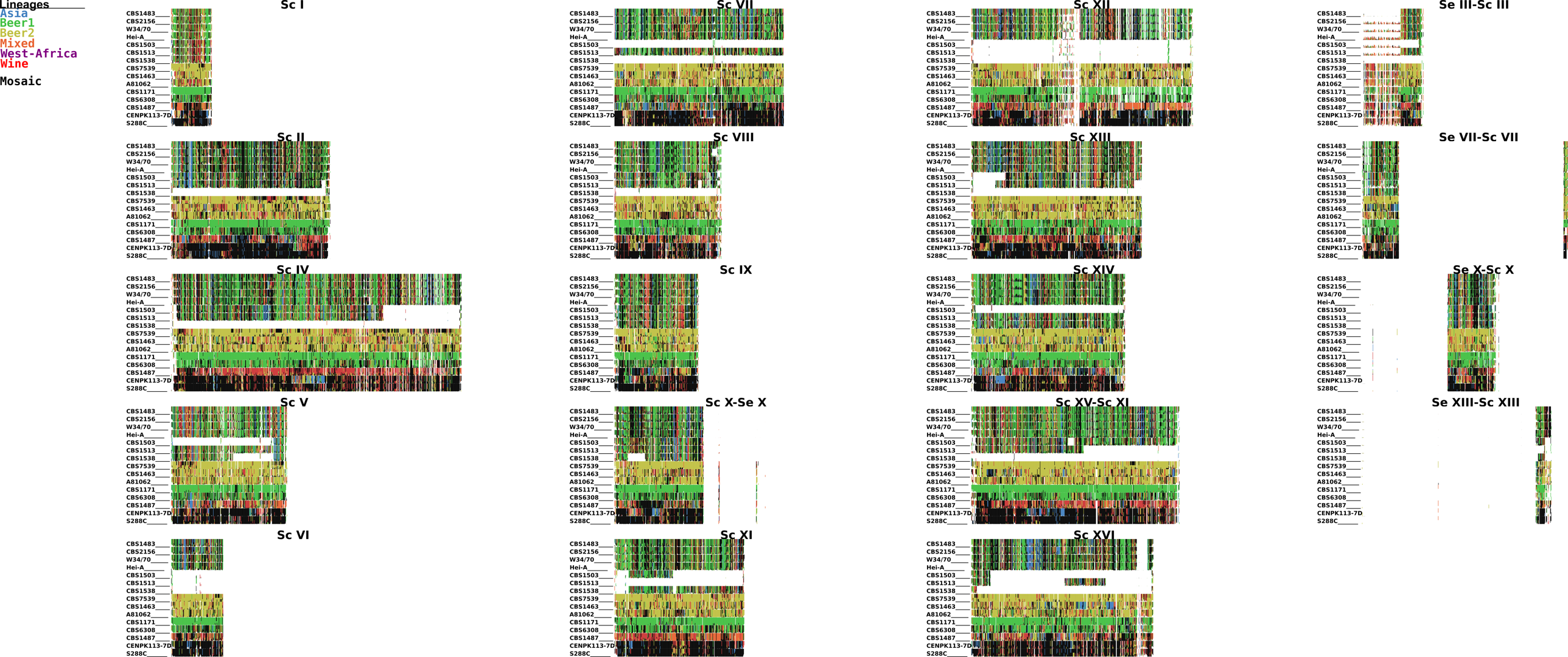

Supplement: Supplementary file 6 — Additional file 6: Figure S5. A more complete version of the similarity profiles of the S. cerevisiae (sub-)genomes of various Saccharomyces strains as shown in Fig. 4. Each S. cerevisiae chromosome of the CBS 1483 assembly was partitioned in non-overlapping sub-regions of 2 Kbp. The colors represent the most similar lineages based on k-mer similarity of 157 S. cerevisiae strains from Gallone et al [62]: Asia (blue), Beer1 (green), Beer2, (gold), Mixed (orange), West-Africa (purple), Wine (red). Mosaic strains are shown in black and ambiguous or low-similarity sub-regions in white. Similarity patterns are shown for the Group 2 S. pastorianus strains CBS 1483, CBS 2156, WS34/70 and Hei-A, for the Group 1 S. pastorianus strains CBS 1503, CBS 1513 and CBS 1538, for S. cerevisiae ale-brewing strains CBS 7539, CBS 1463, A81062, CBS 1171, CBS 6308 and CBS 1483, and for S. cerevisiae laboratory strains CEN.PK113-7D and S288C. [file 12864_2019_6263_MOESM6_ESM.png]

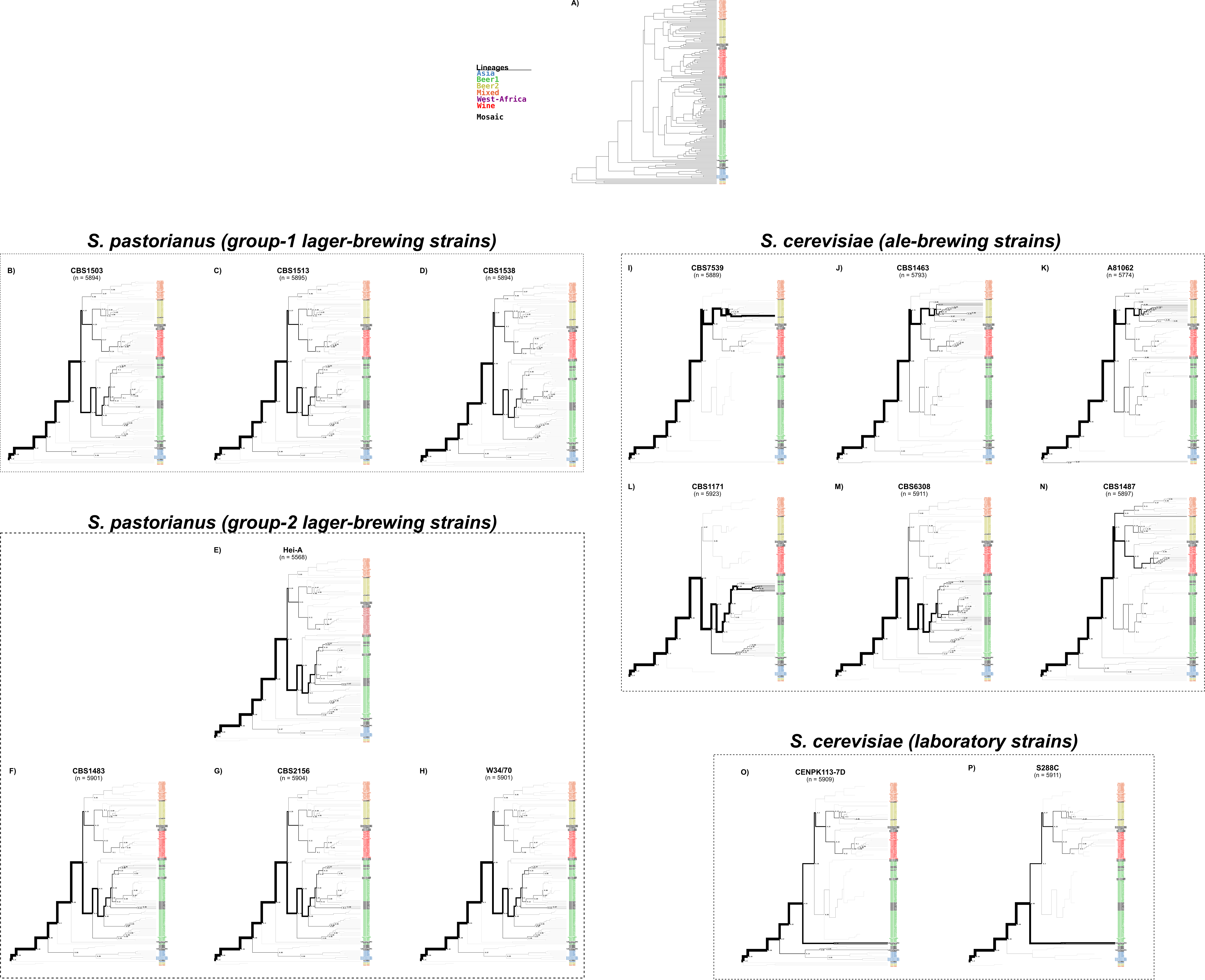

Supplement: Supplementary file 7 — Additional file 7: Figure S6. More complete version of the tree tracing of Fig. 5. Tree-tracing of the genome-scale similarity across the S. cerevisiae (sub-)genomes of various Saccharomyces strains, as determined using Alpaca. The frequency at which a genome from the reference data set of 157 S. cerevisiae strains from Gallone et al [62] was identified as most similar for a sub-region of the CBS 1483 genome is depicted. The reference dataset is represented as a population tree, upon which only lineages with similarity are indicated with a thickness proportional to the frequency at which they were found as most similar. In addition to the genomes of CBS 1513, CBS 1483, CBS 7539 and CBS 1171, the tree-tracing figures of S. pastorianus Group 1 strains CBS 1503 and CBS 1538, of S. pastorianus Group 2 strains CBS 2156, WS34/70 and Hei-A, and of S. cerevisiae strains CBS 1463, A81062, CBS 6308, CBS 1487, CEN.PK113-7D and S288C are shown. [file 12864_2019_6263_MOESM7_ESM.png]

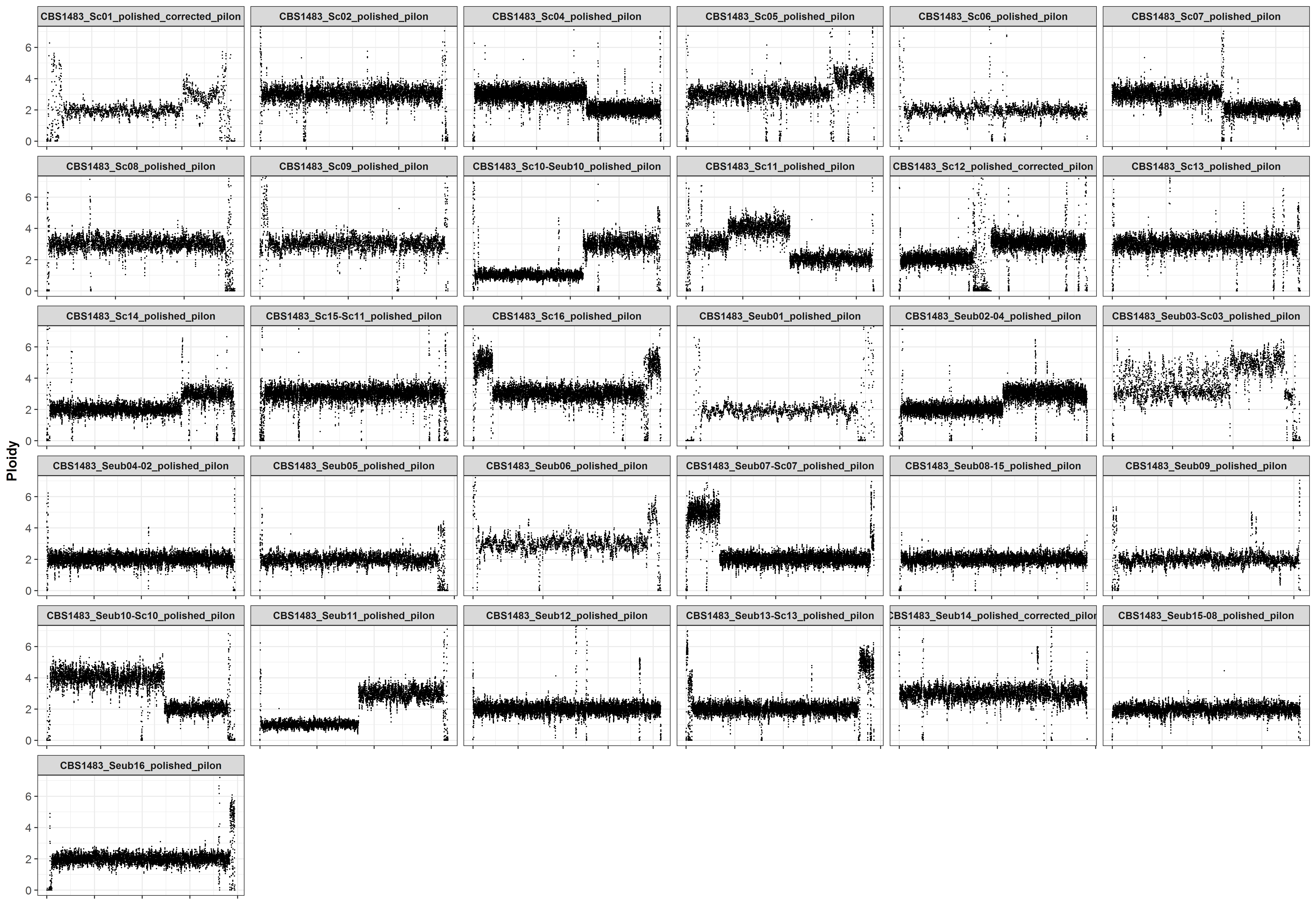

Supplement: Supplementary file 8 — Additional file 8: Figure S7. The ploidy estimates for each chromosome of the S. pastorianus isolate of the Heineken A-yeast® lineage based on alignment of short-read data to the chromosome-level assembly of CBS 1483. [file 12864_2019_6263_MOESM8_ESM.png]
