# Supplementary material for: Chromosome level assembly and comparative genome analysis confirm lager-brewing yeasts originated from a single hybridization
Source: BMC Genomics. 2019 Dec 2;20:916. doi: 10.1186/s12864-019-6263-3 (PMC6889557; doi:10.1186/s12864-019-6263-3)
Supplement: Supplementary file 9 — Additional file 9: Table S1. Whole genome sequencing data generated in this study, under NCB Accession PRJNA522669. [file 12864_2019_6263_MOESM9_ESM.docx]

| Sample | Species | Type | Coverage |
| --- | --- | --- | --- |
| CBS 1483 | *S. pastorianus* | Oxford Nanopore MinION | 72x |
| CBS 1483 | *S. pastorianus* | Illumina | 159x |
| Hei-A | *S. pastorianus* | Illumina | 162x |
| CDFM21L.1 | *S. eubayanus* | Illumina | 73x |
