# Supplementary material for: Chromosome level assembly and comparative genome analysis confirm lager-brewing yeasts originated from a single hybridization
Source: BMC Genomics. 2019 Dec 2;20:916. doi: 10.1186/s12864-019-6263-3 (PMC6889557; doi:10.1186/s12864-019-6263-3)
Supplement: Supplementary file 10 — Additional file 10: Table S2. Homozygous sub-regions of the S. cerevisiae sub-genome used for the SNP-based phylogenetic analysis. [file 12864_2019_6263_MOESM10_ESM.docx]

| Chromosome | Start | End | Size (bp) |
| --- | --- | --- | --- |
| Sc02 | 347,814 | 811,290 | 463,476 |
| Sc04 | 717,861 | 1,479,484 | 761,623 |
| Sc05 | 152,336 | 580,259 | 427,923 |
| Sc07 | 10,001 | 499,577 | 489,576 |
| Sc09 | 10,001 | 291,745 | 281,744 |
| Sc11 | 10,001 | 358,714 | 348,713 |
| Sc13 | 352,974 | 862,991 | 510,017 |
| Sc14 | 10,001 | 561,041 | 551,040 |
| Sc15-Sc11 | 10,001 | 163,161 | 153,160 |
